# Computationally designed Spike antigens induce neutralising responses against the breadth of SARS-COV-2 variants

**DOI:** 10.1038/s41541-024-00950-9

**Published:** 2024-09-09

**Authors:** Sneha Vishwanath, George William Carnell, Martina Billmeier, Luis Ohlendorf, Patrick Neckermann, Benedikt Asbach, Charlotte George, Maria Suau Sans, Andrew Chan, Joey Olivier, Angalee Nadesalingam, Sebastian Einhauser, Nigel Temperton, Diego Cantoni, Joe Grove, Ingo Jordan, Volker Sandig, Paul Tonks, Johannes Geiger, Christian Dohmen, Verena Mummert, Anne Rosalind Samuel, Christian Plank, Rebecca Kinsley, Ralf Wagner, Jonathan Luke Heeney

**Affiliations:** 1 Lab of Viral Zoonotics, Department of Veterinary Medicine, University of Cambridge, Madingley Road, Cambridge, CB3 0ES United Kingdom; 2 Institute of Medical Microbiology and Hygiene, University of Regensburg, Regensburg, Germany; 3 Viral Pseudotype Unit, Medway School of Pharmacy, The Universities of Kent and Greenwich at Medway, Chatham, United Kingdom; 4 MRC-University of Glasgow Centre for Virus Research, Glasgow, United Kingdom; 5 ProBioGenAG, Berlin, Germany; 6 Ethris GmbH, Semmelweisstraße 3, 82152 Planegg, Germany; 7 DIOSynVax Ltd, University of Cambridge, Cambridge, United Kingdom; 8 Institute of Clinical Microbiology and Hygiene, University Hospital Regensburg, Regensburg, Germany

**Keywords:** DNA vaccines, RNA vaccines

## Abstract

Updates of SARS-CoV-2 vaccines are required to generate immunity in the population against constantly evolving SARS-CoV-2 variants of concerns (VOCs). Here we describe three novel in-silico designed spike-based antigens capable of inducing neutralising antibodies across a spectrum of SARS-CoV-2 VOCs. Three sets of antigens utilising pre-Delta (T2_32), and post-Gamma sequence data (T2_35 and T2_36) were designed. T2_32 elicited superior neutralising responses against VOCs compared to the Wuhan-1 spike antigen in DNA prime-boost immunisation regime in guinea pigs. Heterologous boosting with the attenuated poxvirus - Modified vaccinia Ankara expressing T2_32 induced broader neutralising immune responses in all primed animals. T2_32, T2_35 and T2_36 elicited broader neutralising capacity compared to the Omicron BA.1 spike antigen administered by mRNA immunisation in mice. These findings demonstrate the utility of structure-informed computationally derived modifications of spike-based antigens for inducing broad immune responses covering more than 2 years of evolved SARS-CoV-2 variants.

## Introduction

Since human-to-human transmission of SARS-CoV-2 began to spread in late 2019, the virus began to accumulate mutations with different effects on its interaction with the host. As humans began to acquire immunity by vaccination and/or infection, virus variants evolved that were able to escape pre-existing human immune responses and continued to circulate^1^. In addition to the evolution of SARS-CoV-2 in humans, the virus infected other mammals such as mink, cat, dogs, and certain species of deer^2^. Cross-species infections of SARS-CoV-2 contributes to the rate of evolution, fitness, and immune escape features that may enable future SARS-CoV-2 variant epidemics when they re-infect humans^3,4^. Since late 2020, many variants of concern (VOCs) have been reported, beginning with the Alpha, Beta, Gamma, Delta, and the variants of the Omicron lineage. It is primarily the evolution of the spike protein that enabled immune escape and evasion. The emergence of adaptive mutations in the spike protein strongly impacts host tropism and viral transmission^1,5^. Facing an increasingly immune population, immunological escape from host immunity is now a feature of SARS-CoV-2 variants that facilitates the global spread in the immune human population^6^.

With the emergence of each subsequent variant of concerns (VOCs) or sub-variant, there has been a declining neutralising activity of antibodies induced by first generation vaccines based on SARS-CoV-2 Wuhan-1 (Wu-Hu-1) strain. Out of these, Delta, and Omicron sub-variants have been reported with higher transmission rates and immune escape from both naturally as well as vaccine acquired immunity^7–9^. This has necessitated continuous update of SARS-CoV-2 vaccines to match the prevalent circulating strains. Leading COVID-19 licenced vaccine manufacturers have adapted their vaccine to omicron lineage and administered either as a monovalent vaccine or a bivalent vaccine^10–12^. Bivalent vaccines were introduced to generate a broader response covering both the original Wu-Hu-1 strain and recent Omicron lineage, however they have since been shown to be inferior to boosting by single antigen BA.1/BA.5 based vaccine^11,12^. It is suggested that this is due to immune imprinting by the first Wu-Hu-1 spike antigen sequence that was first used by all vaccine manufacturers. Monovalent Spike vaccines have since been recommended over bi-valent vaccines by WHO (https://www.who.int/emergencies/diseases/novel-coronavirus-2019/covid-19-vaccines). Since early 2022, rapid evolution of SARS-CoV-2 occurred in part due to recombination of circulating variants and the antigenic changes selected in partially immune hosts^6^. The Omicron lineage expanded, facilitated by multiple strains dominant in different geographic locations and populations with different SARS-CoV-2 immunity. Under such circumstances of multiple dominant strains, adapting the vaccine to match a single strain or dominant VOC is a suboptimal approach to provide sufficient protective immunity in the global community. Indeed, the situation may acquire similarities with the annual Influenza vaccine field where wild-type strain selection causes variable vaccine efficaciousness from year to year^13^.

Given the emergence of multiple VOCs and different VOCs being dominant at different geographical locations, we hypothesised that vaccine candidates expressing diverse epitopes would be more effective than current practice of using a single or bivalent wild-type strain from the past season. Utilising publicly available sequence data, we developed three computationally designed Spike antigens. One pre-Delta (T2_32), and two different post-Gamma (T2_35 and T2_36) designs were taken forward for immunogenicity studies in small animals. The design - T2_32 and the designs - T2_35, and T2_36 was designed independently at the start of Delta wave and Omicron wave respectively. The T2_32 antigen was first compared with the Wu-Hu-1 based Spike antigen in Guinea pigs using DNA-MVA platform. Subsequently these Spike antigens were delivered by mRNA vaccine to naïve BALB/c mice. We compared the antigens against the wild-type sequence of the Wu-Hu-1 and Omicron BA.1 Spike antigen in mice. Importantly all these novel Spike vaccine antigens demonstrated broader neutralisation profiles as compared to wild-type antigens. The superiority of neutralisation breadth against past and future VOCs that subsequently emerged, more than a year following their original design, underscores the importance of this class of designed vaccine antigens for inclusion as next generation COVID-19 booster vaccine candidates given the superiority in neutralisation breadth compared to wild-type SARS-CoV-2 strains. This data suggests that spike-based antigens expressing multiple epitopes may provide better neutralising titre against existing and emerging VOCs than vaccines using wild-type SARS-CoV-2 VOCs.

## Results

### In-silico design of the antigens

**Fig. 1:**
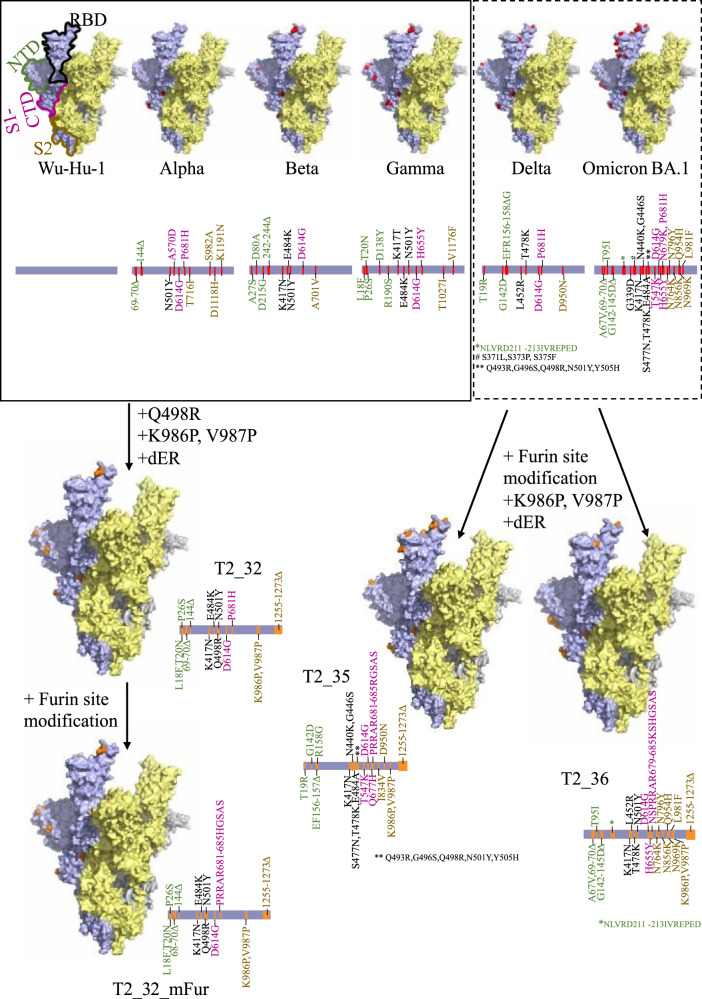
In-silico design of Spike antigens. Surface representation of the extra-virion region of the Spike protein of SARS-CoV-2. The three subunits are coloured in pale yellow, pale blue, and grey. The structural domains – N-terminal domain (NTD), receptor binding domain (RBD), C-terminal domain of the S1 region (S1-CTD) and the stalk region (S2) are highlighted by green, black, magenta, and yellow-brown outlines respectively. The mutations reported in different variants are coloured as red sphere in the surface representation and indicated by red lines in the linear representation. The mutations introduced in the spike vaccine antigens are coloured as orange spheres in the surface representation and indicated by orange lines in the linear representation for T2_32, T2_35, T2_36 and T2_32_mFur. The surface representation was generated and rendered using PyMol^51^ using PDB id. 7ZR9^52^.

The structure of the spike protein of SARS-CoV-2 can be antigenically divided into three distinct regions, the N-terminal domain (NTD), the receptor binding domain (RBD) including the C-terminal domain of S1 subunit (S1-CTD), and the stalk (S2) region. The RBD possesses most of the experimentally characterised epitopes, followed by the NTD and the stalk. The frequent emergence of multiple mutations in the RBD and NTD of the SARS-CoV-2 VOCs suggest that these epitopes are important targets for protective neutralising antibodies. Our computational antigen design approach for the SARS-CoV-2 Spike involved mapping the observed mutations that characterised the VOCs - Alpha, Beta, and Gamma, onto the spike protein of Wu-Hu-1 as a scaffold. We then mapped these mutations as epitope regions, for instance using the epitope information from IEDB^14^ database with those defined as immunodominant epitopes using the peptides reported by Lu et al. ^15^. All the mutations in immunodominant regions were labelled with the VOC it has been observed in and the region it corresponds to in the spike protein. Beta and Gamma variants have the same mutations in the immunodominant region of the RBD (E484K, N501Y) except K417N/T. In addition to the RBD region, the Gamma variant also has mutations in the reported immunodominant region of NTD (L18F, T20N, P26S). As mutations in Gamma variant was reported in two immunodominant regions, set of mutations reported in NTD of the Gamma variant were included in this design. Based on the clustering of mutations – sets of NTD mutations reported in for Alpha (69HΔ, 70VΔ, 144YΔ**)** and Gamma (L18F, T20N, P26S) variants; with RBD mutations reported for Beta (K417N, E484K) VOC; and S1-CTD mutations reported in Alpha (P681H) variants, along with the common N501Y and D614G mutations –were included in the different designs. The Q498R mutation reported by Schreiber et al was also included due to its defined potent role in immune escape and high affinity mutation studied by in-vitro evolution^16^. This mutation was later observed in the Omicron lineage (late 2021). In addition, K986P^17,18^, V987P^17,18^, and deletion of 19 amino acids from C-terminal (dER) were introduced to stabilise the antigen - **T2_32** (Fig. 1). Deletion of 19 amino acid from the C-terminal region was reported to increase the expression of the spike protein on the surface of an infected (or transfected) cell in comparison to the full-length Spike protein^19^ resulting in higher levels of antigen expression. This antigen was evaluated in DNA-MVA prime-boost regime in Guinea pigs so that sufficient antiserum could be collected.

Importantly, in context, T2_32 was designed before the global dominance of Delta and Omicron variants was known. The VOCs Delta and Omicron were both reported to escape pre-existing antibody responses that had been acquired in the human cohorts studied, and were attributable to having accumulated the greatest number of reported mutations at that time^8,20^, we designed another set of antigens as pre-emptive measure to circumvent immune escape and tested these designs as mRNA antigens in mice.

Due to the re-combinatorial nature of the Omicron lineage with multiple distinct mutations in different immunodominant regions of the Spike protein compared to the earlier Delta variant, we undertook to a different approach to the T2_32 Spike design. For the Delta-Omicron designs we combined different sets of mutations on the backbone of Wu-Hu-1 in two distinct ways. In the first instance the NTD and the S2 regions were enriched with the mutation observed in the Delta variant, while the RBD and S1-CTD regions were enriched with the mutations observed in Omicron BA.1 variant - **T2_35** (Fig. 1). For the second Delta-Omicron design - **T2_36** Fig. 1), the NTD and the S2 regions were enriched with the mutation observed in the Omicron BA.1 variant while the RBD and S1-CTD regions were enriched with the mutations observed in Delta variant. This strategy was to ensure all the important immunodominant regions are represented for majority of the VOCs reported up to Dec. 2021 if future recombination between VOCs of past and present may occur. In addition to all the other stabilising mutations in the T2_32 design, the Furin cleavage site was knocked-out (682RRAR to GSAS)^17^ in the two Omicron generation antigen designs. In addition, mutations Q677H, and I834V were introduced in T2_35. Q677H mutation was predicted to increase viral infectivity and neutralisation escape and were observed in many of the variants circulating between February 2021 and December 2021^21,22^. I834V was observed in some of the Delta variants circulating in Asia^23,24^ and hence were included in the design. The K417N mutation was further introduced in T2_36. Notably a Furin cleavage site modified version of T2_32 (henceforth referred as T2_32_mFur) was also generated for comparison in mRNA platform.

### Spike vaccine antigen (T2_32) delivered by DNA and MVA in Guinea pigs

**Fig. 2:**
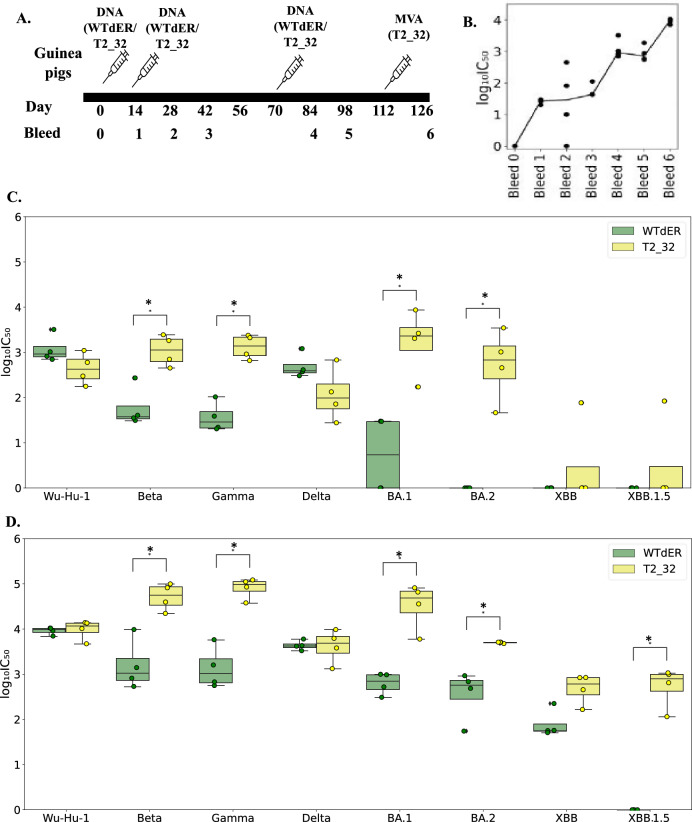
Immunogenicity of T2_32 in Guinea pigs. **A** Immunisation and bleeding schedule in Guinea pigs. **B** Distribution of the neutralisation titres against Wu-Hu-1 pseudotype on immunisation with WTdER. The x-axis represents the bleed number, and the y-axis represents the log_10_(IC_50_) values. **C** Distribution of the neutralisation titres at bleed 4 against Wu-Hu-1 and the VOCs; Beta, Gamma, Delta, BA.1, BA.2, XBB, XBB.1.5. The x-axis represents the pseudoviruses tested for neutralisation, and the y-axis represents the log_10_(IC_50_) values. **D** Distribution of the neutralisation titre of bleed 6 against Wu-Hu-1 and VOCs – Beta, Gamma, Delta, BA.1, BA.2, XBB, XBB.1.5. The x-axis represents the pseudoviruses tested for neutralisation, and the y-axis represents the log_10_(IC_50_) values. The boxplots are colour coded according to vaccines. The boxes represent the quartiles (25th, 50th and 75th percentiles) of the distribution, and the whiskers represent the minimum and maximum of the distribution (excluding outliers) and the fliers represented as filled circle represent the outliers. Mann–Whitney U test is used as statistical significance test in all the plots (*p* value: * ≤0.05, **<0.01, *** ≤ 0.001). The distributions that are not statistically significant are not labelled in the plot. *n* *=* 4 for C and D.

As an initial proof of concept, we first evaluated our first spike design, T2_32 in Guinea pigs using a DNA prime, MVA boost immunisation protocol. Guinea pigs were immunised thrice with the antigens –T2_32, and the dER version of Wu-Hu-1 spike (WTdER) in a pEVAC plasmid and boosted once with MVA.CR19 expressing T2_32 (Fig. 2A). The neutralising titres were longitudinally analysed for WTdER against pseudoviruses (PVs) expressing Wu-Hu-1 spike. The neutralising antibodies titres peaked first by the 4th bleed following three immunisations and peaked again at bleed 6 following MVA boosting (Fig. 2B). The neutralising titre against the VOCs and the Wu-Hu-1 strain were measured for these bleeds (Fig. 2C, D).

Both the antigens induced neutralising titres against Beta, Gamma, and Delta variants, after three DNA immunisations (Fig. 2C). Higher neutralising titres against BA.1 and BA.2 variants were observed only in Guinea pigs immunised with T2_32 (Fig. 2C). Against the recent variants – XBB and XBB.1.5 variants, only one of the guinea pigs immunised with T2_32 generated neutralising titre (Fig. 2C). Except against Wu-Hu-1 and Delta strain, T2_32 generated at least a log higher titre than the WTdER antigen. For both Wu-Hu-1 and Delta strains, the titres were comparable for both the antigen. As higher titres were observed in the group immunised with T2_32 and to study the influence of heterologous boost on background of vaccination by Wu-Hu-1 strain, we boosted both the group of guinea pigs with heterologous vector – MVA expressing T2_32. MVA has been shown as a promising heterologous boost to DNA, resulting in increased neutralising titres^25^. On boosting with MVA expressing T2_32, higher neutralising titres of at least a log-fold for both groups of guinea pigs across the entire VOC panel (Fig. 2D) was observed. Most importantly, significantly higher titres were observed for three of the Omicron variants – BA.1, BA.2, and XBB for both the groups of guinea pigs, as well as significant high titres were for XBB.1.5 in groups primed with T2_32. Taken together, these results show that our T2_32-design is far superior to Wu-Hu-1 based Spike antigens and proved to be future proofed to many of the VOCs when administered as boost.

### Spike vaccine antigens (T2_35, T2_36, and T2_32_mFur) delivered by mRNA in mice

**Fig. 3:**
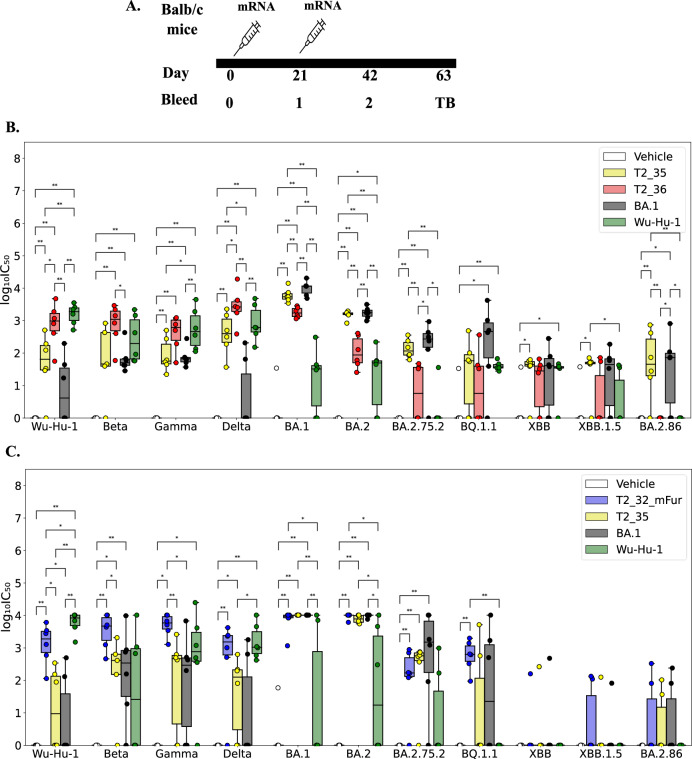
Comparison of breadth of neutralisation of T2_35, T2_36, and T2_32_mFur in mRNA immunised mice. **A** Immunisation and bleed schedule in mice. **B**, **C** Distribution of the neutralisation titres of terminal bleed against Wu-Hu-1 and VOCs. The x-axis represents the pseudoviruses tested for neutralisation, and the y-axis represents the log_10_(IC_50_) values. The boxplots are colour coded according to vaccines. The boxes represent the quartiles (25th, 50th and 75th percentiles) of the distribution, and the whiskers represent the minimum and maximum of the distribution (excluding outliers) with outliers are represented as filled circles. Mann–Whitney U test is used as statistical significance test in all the plots (*p* value: * ≤0.05, **<0.01, *** ≤ 0.001). The distributions that are not statistically significant are not labelled in the plot. *n* *=* 6 for **B** and **C**.

While the T2_32 Guinea pig studies were ongoing, omicron variants became globally dominant in the human population. Omicron variants were reported to show higher resistance to sera from infected and/or vaccinated individuals as well as majority of the known therapeutic antibodies^26^. As a pre-emptive measure to circumvent immune escape by vaccines we designed two additional antigens, T2_35 and T2_36 and formulated them for mRNA delivery. The dER version of Omicron BA.1 and Wu-Hu-1 sequence were used as comparison controls in this immunogenicity study. A 21 day mRNA immunisation regime was followed by bleeds at 42 and 63 days (Fig. 3A). Notably the Omicron generation – T2_35 and T2_36 spike antigens were able to induce broad neutralising responses against Alpha, Beta, Gamma, Delta, and many of the important Omicron/Omicron-like lineages (Fig. 3B, C). Interestingly while both Omicron generation antigens generated a robust neutralising titre against all the VOCs, there was a distinctly different pattern against the VOCs that occurred before and after the first Omicron variants appeared. T2_35 generated higher neutralising titres against VOCs from BA.1 forward into the later omicron sub-lineages, while T2_36 generated higher neutralising titres to pre-omicron VOCs (Alpha through to Delta Fig. 3B, C). This difference in the neutralising patterns against the VOCs could be explained on the different sets of mutations used in different regions in these two designs. T2_35 is enriched with mutations observed in Omicron BA.1 variant in the RBD region while T2_36 is enriched with mutations observed in Delta variant in the same RBD region. This data confirms previous reports on immunodominance epitopes in the RBD over the NTD or S2 regions.

Interestingly, the T2_35 candidate generated neutralising titres comparable to BA.1 antigen against all the Omicron variants, but a significantly higher titres to Delta variant than BA.1 antigen. Although the T2_36 generated lower neutralising titres against Omicron variants compared to BA.1 antigen, it significantly induced higher neutralising antibody titres against non-Omicron VOCs as compared to BA.1 antigen.

We further asked if the Furin cleavage site modified T2_32 (T2_32_mFur) was as immunogenic as compared to the antigens - T2_35, dER versions of Omicron BA.1 and Wu-Hu-1. Interestingly the T2_32_mFur demonstrated superior immunogenic responses to all the VOCs tested compared to Wu-Hu-1 antigen, with comparable immunogenicity to BA.1 antigen (Fig. 3C). The immunogenic response of T2_32_mFur against the Omicron sub-lineage could be attributed to the common mutations in some of the immunodominant regions. For example, HV69-70Δ is common between T2_32_mFur and BA.2.86, BF.7, BQ.1.12, and BQ.1.1; while Y144Δ is common between XBB.1.5, BA.2.86, and T2_32mFur. While the K417N, Q496R, N501Y, and P681H mutations in T2_32_mFur were observed in all the tested Omicron lineage.

Though neutralising titres have been observed against many of the VOCs by T2_35 and T2_32_mFur antigens, it must be noted that the titres are lower for current VOCs such as BQ.1.1, XBB.1.5 etc., than the VOCs such as Delta, BA.2. The decrease in titre is due to higher number of diverging mutations in these VOCs. In view of this observation, the designs may need to be updated for highly divergent VOCs or any future variant with completely different set of mutations at immunodominant epitopes. Overall, the data supports the broader neutralisation profile of the designed spike antigens, even neutralising the VOCs detected more than two-years after the first design.

## Discussion

Despite the critical role that currently licensed vaccines have had in mitigating the severity of COVID-19 disease, the continued emergence of SARS-CoV-2 variants with their increasingly complex immune evasion characteristics, suggest that alternative vaccine booster strategies are needed. Furthermore because of strong selective pressures posed by existing immune responses, generated either by natural infection or vaccination, emerging variants of SARS-CoV-2 retain a high probability of reinfection. Although boosting of the immune response with the original, historical Spike antigen increases antibody titres^27^, they are ineffective against future immune evasive variants such as the XBB and BQ.1 lineage^27–29^. In view of these challenges, the WHO has recommended the COVID vaccine antigen to match the pre-dominant circulating variant or strain^30^. Over the four years of the evolution of SARS-CoV-2 variants, the vaccine composition has been updated multiple times with wild-type variants in an effort to match the prevalent circulating strains. This is intended to update the immune landscape of the human population with the evolving landscape of emergence of SARS-CoV-2 variants. Though the updated vaccine composition has resulted in the increased titre towards the variants in circulation, they have proved to have lower titres against the emerging VOCs^27^. Moreover, the problem of “annually” immunising with wild-type viral Spikes vaccines from viral strains that have (even recently) occurred in the past, may not provide effective protection against the circulating variants due to mismatch between the variants circulating across the globe, a problem observed with Influenza vaccines each year. Notably in the fall 2023 the COVID-19 vaccine booster consisted of monovalent XBB.1.5, which was first detected on the 22^nd^ of October 2022 (https://www.ecdc.europa.eu/en/news-events/update-sars-cov-2-variants-ecdc-assessment-xbb15-sub-lineage). The current practice is reactive and behind the curve. The time between identification of VOC XBB.1.5, and the time the vaccine had been manufactured and distributed was approximately a year, giving the targeted vaccine strain significant time to circulate globally and acquire new immune evasive characteristics in partially immune populations. An early approach that was adopted by the industry to increase the breadth of the vaccines was introduction of bivalent vaccines. But bivalent vaccines were soon demonstrated to be inferior to single antigen-based vaccines, due to the impact of immune imprinting (stronger boosting of immune memory responses to previously recognises SARS-CoV-2 epitopes). Hence, higher titres were observed against the original Wu-Hu-1 strain in comparison to titres against new BA.1 and BA.5 variants^11,12^. Therefore, in the face of the continuously evolving landscape of vaccine evasive SARS-CoV-2 variants, we developed a broader, pro-active strategy using novel synthetic antigens with marked antigenic properties that incorporate critical mutations providing superior breadth of immune responses against future variants.

Advances in genomic tracking of viral epidemics, as witnessed during the COVID-19 pandemic, have enabled the real-time assessment of vaccines to neutralise emerging viral variants. Here we leverage the patterns of pathogen genomics and vaccine escape to design next generation vaccine antigens for COVID-19 with neutralisation capacity against multiple lineages of variants of SARS-CoV-2. We demonstrate that these novel spike-based antigens that incorporate critical mutations observed in the evolution of SARS-CoV-2 VOCs to target multiple lineages of SARS-CoV-2 variants. We demonstrated that boosting of animals previously immunised with Wu-Hu-1 spike antigen, with our T2_32 vaccine antigen, significantly increased the titres against all the tested VOCs. This observation validated the utility of a non-wild-type booster antigen which was able to expand the breadth of the immune response encompassing many of the future variants. The observed immunogenicity of T2_32 validated our rationale that novel spike antigens consisting of multiple epitopes from VOCs will provide greater vaccine efficacy against future VOCs compared to current variant/strain-selection approaches. In practise, this observation is important in the context of utilizing new designs for populations that have been primed and boosted with first generation COVID-19 vaccines.

During these studies the Omicron lineages arose and became dominant in the human population. The Omicron lineage accrued multiple mutations in both the NTD and RBD regions of the spike protein and was reported to escape previous existing COVID-19 vaccine immunity^8,20^. As a pre-emptive measure to counter immune escape we designed two additional antigens (T2_35 and T2_36) and delivered them as LNP formulated mRNA to mice. Both the candidates T2_35 and T2_36 generated a neutralising response against all the tested VOCs representing evolved variants over more than 2 years. Notably the immunogenicity profile of T2_36 against the Omicron lineage in comparison to Wu-Hu-1 spike antigen supports the role of antibodies generated against the NTD/stalk region in broader immune responses against VOCs. Comparison of the breadth and potency induced in mice across the VOCs tested for, T2_35 was found to have the best immunological profile, followed by T2_36 over the wild type BA.1 Spike vaccine. As we observed neutralising response against some of the variants from Omicron lineage by T2_32 in DNA-MVA regime, we further tested the immunogenicity of Furin cleavage site modified version of T2_32 (T2_32_mFur) delivered by mRNA immunisation in mice. Interestingly the T2_32_mFur showed good neutralisation titre against many variants of Omicron lineages with titres comparable to BA.1 and T2_35. Taken together, the observation of a significantly broader neutralisation profile of the designs – T2_32, T2_35, and T2_36 antigens in comparison to the wild-type Spike – Wu-Hu-1 and BA.1 antigens, validated the superiority of computationally designed antigens over a single wild-type VOC Spike based antigens.

Novel SARS-CoV-2 vaccines have been developed and tested by other and our groups, including the use of nanoparticles expressing multipe wild-type RBDs from different Sarbecoviruses lineages^31^, single antigen targeting multiple sarbecoviruses^32^, as well as consensus-based approach to variant proof designs^33^ that have shown promising pre-clinical broad-neutralisation of VOCs. Of these approaches, our Digital Immune Optimised Synthetic Vaccine (DIOSynVax) antigen designs are related but computationally different to the consensus based SPAN approach^33^, which have also observed broader neutralisation ability of their design in comparison to the prototype wild-type based vaccines. The SPAN approach is based on generation of consensus sequence. In contrast, our DIOSynVax approach uses specific and unique immunodominant mutations from VOCs to construct novel Spike structures. In conclusion, the Digital Immune Optimised Synthetic multi-epitope vaccine antigen designs generated broad neutralising responses covering over 2 years of evolved SARS-CoV-2 variants, offering a superior vaccine antigen strategy to the current practice of using wild-type vaccine evasive spike antigens as booster vaccines.

## Methods

### In-silico design of the vaccine antigens

A consensus sequence was generated for spike protein of VOCs - Alpha, Beta, and Gamma using the sequences deposited in NCBI Virus (Feb. 2021)^34^. Multiple sequence alignment (MSA) was generated for Wu-Hu-1 (NC_045512.2), Alpha, Beta, and Gamma using MAFFT algorithm^35^. The mutated amino acids for each VOCs were identified. Information on epitope residues were retrieved from the Immune Epitope DataBase (IEDB)^14^ and mapped along the length of the MSA to identify the mutations associated with immune inducing region of the spike protein. Further, epitope regions were classified as immunodominant region based on literature^15^. For further analyses, only mutations occurring in the immunodominant epitope region of the spike protein were considered, as they would contribute maximally to immune escape. Each mutation in immunodominant epitope were assigned to the four structural domains – N-terminal domain (NTD), receptor binding domain (RBD), C-terminal domain of S1 (S1-CTD), and stalk (S2) region and labelled with the VOCs it is observed in. The set of mutations from the NTD of the Alpha and Gamma variants, the set of mutations from the RBD region of the Beta, and the set of mutations from the S1-CTD of the Alpha variant were introduced on the backbone of the Wu-Hu-1 strain. Further mutations - K986P^17,18^, V987P^17,18^ and Q498R^16^ and deletion of 19 amino acids from C-Terminal of Spike protein were introduced to the final design -**T2_32** (Fig. 1).

Using the similar design protocol, two designs – **T2_35** and **T2_36** were generated using MSA including the consensus Delta and Omicron BA.1 (Dec. 2021). Sets of mutations from the NTD and S2 domain of Delta variant and set of mutations from the RBD and S1-CTD of Omicron BA.1 were introduced on the backbone of Wu-Hu-1. In addition, mutations Q677H, I834V, K986P^17,18^, V987P^17,18^, replacing Furin cleavage site with GSAS motif^17^, and deletion of 19 amino acids from C-Terminal of Spike protein were introduced to the final design – **T2_35** (Fig. 1). Sets of mutations from the NTD and S2 domain of Omicron BA.1 variant and set of mutations from the RBD and S1-CTD of Delta were introduced on the backbone of Wu-Hu-1. In addition, mutations K417N, K986P^17,18^, V987P^17,18^, replacing Furin cleavage site with GSAS motif^17^, and deletion of 19 amino acids from C-Terminal of Spike protein were introduced to the final design – **T2_36** (Fig. 1).

The structural integrity of the resultant vaccine antigens was checked for by generating homology models using Modeller algorithm^36^. The 19 amino acids from the C-terminal of the spike protein were removed from all the wild-type controls as well. Subsequently, a Furin cleavage site modified version of T2_32 (referred as T2_32_mFur) was also designed.

### Production and transformation of plasmids

Sequences of vaccine designs (T2_32 and Wu-Hu-1) were RNA- and codon-optimised for high level expression in human cells *via* the GeneOptimizer algorithm^37^. These genes were cloned into pEVAC^38^ (GeneArt/Thermofisher, Germany) *via* restriction digestion using kpn1 and not1. Plasmids were transformed *via* heat-shock in chemically competent *E. coli* DH5α cells (Invitrogen 18265-017). Plasmid DNA was extracted from transformed bacterial cultures *via* the Plasmid Mini Kit (Qiagen 12125). The DNA plasmids were purified using the EndoFree Plasmid Mega kit (Qiagen, Hilden, Germany) according to the manufacturer’s instructions. Plasmids were quality controlled by sanger sequencing and quantified using UV spectrophotometry (NanoDrop™ -Thermo Scientific) and assessed for absence of endotoxin.

### Production of MVA

The MVA strain used in this study was MVA-CR19^39^. Recombinant MVA that expresses T2_32 was generated as described previously^25^. Briefly, for homologous recombination adherent CR.pIX cells were infected with MVA-CR19-GFP with different MOIs ranging from 0.5 to 0.006 and transfected with 0.4 μg shuttle plasmid pMVA Trans TK- SARS-CoV-2 T2_32 using Effectene (Qiagen, Germany). After 48 h the lysates were harvested, treated by three freeze-thaw cycles and sonicated. Then, different dilutions of the virus lysates were transferred to a fresh monolayer of AGE1.CR.pIX cells. After 2 h, a semisolid agarose overlay was applied and recombinant MVAs were identified by lacZ-staining using X-Gal (5-bromo-4-chloro-3-indolyl-β-D-galactopyranoside) and isolated with a pipette tip 72 h post infection. Genomic DNA was extracted from the isolated plaques after each agarose plaque purification round using the Quick-DNA Miniprep Kit from Zymo Research according to the manufacturer’s instructions. The correct integration into the TK locus was verified by PCR analysis using primers flanking the TK locus (CTCTCTAGCTACCACCGCAA and ATGCGTCCATAGTCCCGTTC). PCR products were separated on a 1% agarose gel, excised, and the expected sequence was confirmed by Sanger sequencing. The recombinant MVA-CR19 encoding T2_32 was plaque purified for additional three rounds and purified *via* two ultracentrifugation rounds at 20000 rpm at 4 °C using a 35% sucrose cushion.

The resulting recombinant MVA-CR19 T2_32 (MVA T2_32) virus stock was produced in suspension AGE1.CR.pIX cells as described previously^40^. Briefly, cells were seeded in shake flasks in a final volume of 45 mL in a mixture of CD-U7 (Xell) and DMEM (Gibco) (1:1) chemically-defined media, infected with an MOI of 0.1, and incubated in a shaking incubator with 180 rpm, 5 cm amplitude, 37 °C, and 5% CO2 (HT Multitron Cell, Infors AG, Switzerland). The culture was centrifuged 48 h post infection with 1100 × g for 5 min and 35 mL of the supernatant were discarded. The pellet was re-suspendend in 10 mL of the supernatant. This suspension was distributed into 10 vials and sonicated with a Vial Tweeter (Hielscher) for 20 s at 100% cycle and 90% amplitude.The virus titer in the lysate was determined on DF-1 cells using crystal violet staining. The absence of revertant or parental MVA and T2_32 insert identity was confirmed by PCR amplification and Sanger sequencing. Correct expression of T2_32 was confirmed by Western blot (40150-T62, Sino Biological; China) with cell lysates from HEK293 cells harvested 24 h after infection (MOI 2) with MVA T2_32.

### Vaccination experiments in Guinea pigs

Two groups of four seven-week-old female Hartley guinea pigs were purchased from Envigo (Maastricht, Netherlands). Guinea pigs were immunised at 14 days intervals with 200 µg DNA vaccines bearing the antigen gene in the pEVAC vector, administered by intradermal route using the Pharmajet© device in a total volume of 200 µl over the hind legs. A third dose of DNA was administered at day 70 post prime and at day 112 was boosted intramuscular with MVA encoding T2_32 with 2.0E^7^ PFU/dose. Bleeds were taken through the saphenous vein and animals euthanised at the end of experiments using Pentoject under non-recovery anaesthesia.

### Synthesis and packaging of mRNA

mRNA encoding the sequences of the vaccine antigens (T2_35, T2_36, T2_32_mFur, Wuhan-Hu-1, and Omicron BA.1) were synthesised by in vitro transcription (IVT) from linearised plasmid DNA templates using modified nucleotides to generate partial modified mRNAs. IVT was performed for 120 min at 37 °C using T7-RNA polymerase and co-transcriptional capping via Anti-Reverse Cap Analog (ARCA), followed by digestion of the template using DNAse I. After IVT, mRNAs were dephosphorylated for 15 min at 37 °C using alkaline phosphatase and enzymatically polyadenylated for 10-30 min at 37 °C using PolyA polymerase to produce a Poly A tail of approximately 120 nucleotides. Purification steps were performed by precipitation and subsequently formulated in water for injection at a concentration of 1 mg/mL. mRNAs were stored at -80 °C until LNP encapsulation. Each mRNA was LNP encapsulated *via* nanoprecipitation by microfluidic mixing of mRNA in citrate buffer (pH 4.5) with ionizable-, structural-, helper- and polyethylene glycol (PEG) lipids in ethanol, followed by buffer exchange and concentration *via* tangential flow filtration. mRNA LNPs were filtered through a 0.2μm membrane and stored at -20 °C until use. After manufacturing and freezing, the drug product was analytically characterised for particle size ( ≤ 100 nm), particle polydispersity ( ≤ 0.20), encapsulation efficiency ( ≥ 90%), mRNA integrity ( ≥ 90%) and mRNA identity (confirmed length).

### Immunisation of mice

Female 8–10-week-old BALB/c mice (Charles River Laboratories, Kent, United Kingdom) were immunised twice at an interval of 21 days. A total volume of 100 µl of PBS containing 10 µg of lipid encapsulated mRNA encoding the antigens was administered intramuscularly over the two hind legs. The naïve mice group were administered 100 µl of vehicle only control. Bleeds were taken 3 weeks after each immunisation, and a final terminal bleed 6 weeks after the second immunisation under non-recovery anaesthesia.

### Production of lentiviral pseudotypes

Lentiviral pseudotypes were produced by transient transfection of HEK293T/17 cells with packaging plasmids p8.91^41,42^ and pCSFLW^43^ and different SARS-CoV-2 VOC spike-bearing expression plasmids in the pEVAC backbone, using the Fugene-HD (Promega E2311) transfection reagent^44,45^. Supernatants were harvested after 48 h, passed through a 0.45 µm cellulose acetate filter, and titrated on HEK293T/17 cells transiently expressing human ACE-2 and TMPRSS2. Target HEK293T/17 cells were transfected 24 h prior with 2 µg pCAGGS-huACE-2 and 150 ng pCAGGS-TMPRSS2 in a T75 tissue culture flask^46,47^. The sequences of the VOCs spike used for the production of pseudotypes are provided in the supplementary file.

### Pseudotype-based micro-neutralisation assay

Pseudotype-based micro-neutralisation assays (pMN) were performed as described previously^48^. Briefly, serial dilutions of serum were incubated with lentiviral pseudotypes bearing SARS-CoV-2, and SARS-CoV-2 VOC spikes for 1 h at 37 °C, 5% CO_2_ in 96-well white cell culture plates. 1.5 × 10^4^ HEK293T/17 cells transiently expressing human ACE-2 and TMPRSS2 were then added per well and plates incubated for 48 h at 37 °C, 5% CO_2_ in a humidified incubator. Bright-Glo (Promega) was then added to each well and luminescence read after a five-minute incubation period. Experimental data points were normalised to 100% and 0% neutralisation using cell only and virus + cell control wells respectively and non-linear regression analysis performed in GraphPad Prism 9 to produce neutralisation curves and IC_50_ values.

### Statistical analyses

Two-tailed Mann–Whitney U tests were performed for all the pairwise comparisons using the Python sklearn package^49^. All the plots were generated using the Python Matplotlib package and statannotat package^50^.

## Supplementary information

Supplementary Information

## Data Availability

The main data supporting the results in this study are available within the paper. The sequences used for designing the vaccine antigens were retrieved from the publicly available NCBI virus database.

## References

[1] Carabelli, A. M. et al. SARS-CoV-2 variant biology: immune escape, transmission and fitness. *Nat. Rev. Microbiol* **21**, 162–177 (2023). 36653446 10.1038/s41579-022-00841-7 PMC9847462

[2] Tan, C. C. S. et al. Transmission of SARS-CoV-2 from humans to animals and potential host adaptation. *Nat. Commun.* **13**, 2988 (2022). 35624123 10.1038/s41467-022-30698-6 PMC9142586

[3] Divergent SARS-CoV-2 variant emerges in white-tailed deer with deer-to-human transmission - PMC. https://www.ncbi.nlm.nih.gov/pmc/articles/PMC9712111/. 10.1038/s41564-022-01268-9 PMC9712111 36357713

[4] Porter, A. F., Purcell, D. F. J., Howden, B. P. & Duchene, S. Evolutionary rate of SARS-CoV-2 increases during zoonotic infection of farmed mink. *Virus Evol.* **9**, vead002 (2023). 36751428 10.1093/ve/vead002 PMC9896948

[5] Starr, T. N. et al. Deep Mutational Scanning of SARS-CoV-2 Receptor Binding Domain Reveals Constraints on Folding and ACE2 Binding. *Cell* **182**, 1295–1310.e20 (2020). 32841599 10.1016/j.cell.2020.08.012 PMC7418704

[6] Markov, P. V. et al. The evolution of SARS-CoV-2. *Nat. Rev. Microbiol* **21**, 361–379 (2023). 37020110 10.1038/s41579-023-00878-2

[7] Qu, P. et al. Neutralization of the SARS-CoV-2 Omicron BA.4/5 and BA.2.12.1 Subvariants. *N. Engl. J. Med.* **386**, 2526–2528 (2022). 35704428 10.1056/NEJMc2206725 PMC9258774

[8] Hu, J. et al. Increased immune escape of the new SARS-CoV-2 variant of concern Omicron. *Cell Mol. Immunol.* **19**, 293–295 (2022). 35017716 10.1038/s41423-021-00836-z PMC8749347

[9] Lewnard, J. A. et al. Association of SARS-CoV-2 BA.4/BA.5 Omicron lineages with immune escape and clinical outcome. *Nat. Commun.* **14**, 1407 (2023). 36918548 10.1038/s41467-023-37051-5 PMC10012300

[10] Chalkias, S. et al. A Bivalent Omicron-Containing Booster Vaccine against Covid-19. *N. Engl. J. Med* **387**, 1279–1291 (2022). 36112399 10.1056/NEJMoa2208343 PMC9511634

[11] Collier, A. Y. et al. Immunogenicity of BA.5 Bivalent mRNA Vaccine Boosters. *N. Engl. J. Med* **388**, 565–567 (2023). 36630611 10.1056/NEJMc2213948 PMC9847505

[12] Offit, P. A. Bivalent Covid-19 Vaccines — A Cautionary Tale. *N. Engl. J. Med.* **388**, 481–483 (2023). 36630616 10.1056/NEJMp2215780

[13] Krammer, F. The human antibody response to influenza A virus infection and vaccination. *Nat. Rev. Immunol.* **19**, 383–397 (2019). 30837674 10.1038/s41577-019-0143-6

[14] Vita, R. et al. The Immune Epitope Database (IEDB): 2018 update. *Nucleic Acids Res* **47**, D339–D343 (2019). 30357391 10.1093/nar/gky1006 PMC6324067

[15] Lu, S. et al. The immunodominant and neutralization linear epitopes for SARS-CoV-2. *Cell Rep.* **34**, 108666 (2021). 33503420 10.1016/j.celrep.2020.108666 PMC7837128

[16] Zahradník, J. et al. SARS-CoV-2 variant prediction and antiviral drug design are enabled by RBD in vitro evolution. *Nat. Microbiol* **6**, 1188–1198 (2021). 34400835 10.1038/s41564-021-00954-4

[17] Amanat, F. et al. Introduction of Two Prolines and Removal of the Polybasic Cleavage Site Lead to Higher Efficacy of a Recombinant Spike-Based SARS-CoV-2 Vaccine in the Mouse Model. *mBio* **12**, e02648–20 (2021). 33653892 10.1128/mBio.02648-20 PMC8092267

[18] Bos, R. et al. Ad26 vector-based COVID-19 vaccine encoding a prefusion-stabilized SARS-CoV-2 Spike immunogen induces potent humoral and cellular immune responses. *npj Vaccines* **5**, 1–11 (2020). 33083026 10.1038/s41541-020-00243-x PMC7522255

[19] Ujike, M., Huang, C., Shirato, K., Makino, S. & Taguchi, F. The contribution of the cytoplasmic retrieval signal of severe acute respiratory syndrome coronavirus to intracellular accumulation of S proteins and incorporation of S protein into virus-like particles. *J. Gen. Virol.* **97**, 1853–1864 (2016). 27145752 10.1099/jgv.0.000494 PMC5764123

[20] Fan, Y. et al. SARS-CoV-2 Omicron variant: recent progress and future perspectives. *Sig Transduct. Target Ther.* **7**, 1–11 (2022). 10.1038/s41392-022-00997-x PMC9047469 35484110

[21] Zeng, C. et al. Neutralization of SARS-CoV-2 Variants of Concern Harboring Q677H. *mBio* **12**, e02510-21 (2021). 34607452 10.1128/mBio.02510-21 PMC8527387

[22] Niu, X. et al. Isolation and characterization of a SARS-CoV-2 variant with a Q677H mutation in the spike protein. *Arch. Virol.* **168**, 5 (2023). 10.1007/s00705-022-05621-5 PMC9767398 36539656

[23] Jeong, S. et al. Tracking the Genomic Evolution of SARS-CoV-2 for 29 Months in South Korea. *Viruses* **15**, 873 (2023). 37112852 10.3390/v15040873 PMC10142693

[24] CoV-GLUE Lineage. https://cov-glue.cvr.gla.ac.uk/lineage.php?lineage=B.1.617.2.

[25] Carnell, G. W. et al. Glycan masking of a non-neutralising epitope enhances neutralising antibodies targeting the RBD of SARS-CoV-2 and its variants. *Front. Immunol.* **14**, 1118523 (2023). 36911730 10.3389/fimmu.2023.1118523 PMC9995963

[26] Ju, B. et al. Immune escape by SARS-CoV-2 Omicron variant and structural basis of its effective neutralization by a broad neutralizing human antibody VacW-209. *Cell Res* **32**, 491–494 (2022). 35260792 10.1038/s41422-022-00638-6 PMC8902274

[27] Miller, J. et al. Substantial Neutralization Escape by SARS-CoV-2 Omicron Variants BQ.1.1 and XBB.1. *N. Engl. J. Med.* **388**, 662–664 (2023). 36652339 10.1056/NEJMc2214314 PMC9878581

[28] Uraki, R. et al. Antiviral and bivalent vaccine efficacy against an omicron XBB.1.5 isolate. *Lancet Infect. Dis.* **23**, 402–403 (2023). 36773622 10.1016/S1473-3099(23)00070-1 PMC9908083

[29] Yang, J. et al. Low levels of neutralizing antibodies against XBB Omicron subvariants after BA.5 infection. *Sig Transduct. Target Ther.* **8**, 1–12 (2023). 10.1038/s41392-023-01495-4 PMC10279763 37336889

[30] Statement on the antigen composition of COVID-19 vaccines. https://www.who.int/news/item/18-05-2023-statement-on-the-antigen-composition-of-covid-19-vaccines.

[31] Cohen, A. A. et al. Mosaic nanoparticles elicit cross-reactive immune responses to zoonotic coronaviruses in mice. *Science* **371**, 735–741 (2021). 33436524 10.1126/science.abf6840 PMC7928838

[32] Vishwanath, S. et al. A computationally designed antigen eliciting broad humoral responses against SARS-CoV-2 and related sarbecoviruses. *Nat. Biomed. Eng* 1–14 10.1038/s41551-023-01094-2 (2023). 10.1038/s41551-023-01094-2 PMC11839467 37749309

[33] Zhao, Y. et al. Vaccination with Span, an antigen guided by SARS-CoV-2 S protein evolution, protects against challenge with viral variants in mice. *Sci. Transl. Med.* **15**, eabo3332 (2023). 36599007 10.1126/scitranslmed.abo3332

[34] Brister, J. R., Ako-Adjei, D., Bao, Y. & Blinkova, O. NCBI viral genomes resource. *Nucleic Acids Res* **43**, D571–D577 (2015). 25428358 10.1093/nar/gku1207 PMC4383986

[35] Katoh, K., Misawa, K., Kuma, K. & Miyata, T. MAFFT: a novel method for rapid multiple sequence alignment based on fast Fourier transform. *Nucleic Acids Res.* **30**, 3059–3066 (2002). 12136088 10.1093/nar/gkf436 PMC135756

[36] Sali, A. & Blundell, T. L. Comparative protein modelling by satisfaction of spatial restraints. *J. Mol. Biol.* **234**, 779–815 (1993). 8254673 10.1006/jmbi.1993.1626

[37] Raab, D., Graf, M., Notka, F., Schödl, T. & Wagner, R. The GeneOptimizer Algorithm: using a sliding window approach to cope with the vast sequence space in multiparameter DNA sequence optimization. *Syst. Synth. Biol.* **4**, 215–225 (2010). 21189842 10.1007/s11693-010-9062-3 PMC2955205

[38] Del Rosario, J. M. M. et al. Exploiting Pan Influenza A and Pan Influenza B Pseudotype Libraries for Efficient Vaccine Antigen Selection. *Vaccines* **9**, 741 (2021). 34358157 10.3390/vaccines9070741 PMC8310092

[39] Jordan, I. et al. A Deleted Deletion Site in a New Vector Strain and Exceptional Genomic Stability of Plaque-Purified Modified Vaccinia Ankara (MVA). *Virol. Sin.* **35**, 212–226 (2020). 31833037 10.1007/s12250-019-00176-3 PMC7198643

[40] Jordan, I. et al. A chemically defined production process for highly attenuated poxviruses. *Biologicals* **39**, 50–58 (2011). 21237672 10.1016/j.biologicals.2010.11.005

[41] Zufferey, R., Nagy, D., Mandel, R. J., Naldini, L. & Trono, D. Multiply attenuated lentiviral vector achieves efficient gene delivery in vivo. *Nat. Biotechnol.* **15**, 871–875 (1997). 9306402 10.1038/nbt0997-871

[42] Naldini, L. et al. *In Vivo Gene Delivery and Stable Transduction of Nondividing Cells by a Lentiviral Vector*. http://science.sciencemag.org/. 10.1126/science.272.5259.263 8602510

[43] Demaison, C. et al. High-level transduction and gene expression in hematopoietic repopulating cells using a human immunodeficiency [correction of imunodeficiency] virus type 1-based lentiviral vector containing an internal spleen focus forming virus promoter. *Hum. gene Ther.* **13**, 803–813 (2002). 11975847 10.1089/10430340252898984

[44] Sampson, A. T. et al. Coronavirus Pseudotypes for All Circulating Human Coronaviruses for Quantification of Cross-Neutralizing Antibody Responses. *Viruses* **13**, 1579 (2021). 34452443 10.3390/v13081579 PMC8402765

[45] Di Genova, C. et al. Production, Titration, Neutralisation, Storage and Lyophilisation of Severe Acute Respiratory Syndrome Coronavirus 2 (SARS-CoV-2) Lentiviral Pseudotypes. *Bio Protoc.* **11**, e4236 (2021). 34859134 10.21769/BioProtoc.4236 PMC8595416

[46] Hoffmann, M. et al. SARS-CoV-2 Cell Entry Depends on ACE2 and TMPRSS2 and Is Blocked by a Clinically Proven Protease Inhibitor. *Cell* **181**, 271–280.e8 (2020). 32142651 10.1016/j.cell.2020.02.052 PMC7102627

[47] Bertram, S. et al. Influenza and SARS-coronavirus activating proteases TMPRSS2 and HAT are expressed at multiple sites in human respiratory and gastrointestinal tracts. *PLoS ONE* **7**, 1–8 (2012). 10.1371/journal.pone.0035876 PMC3340400 22558251

[48] Carnell, G., Grehan, K., Ferrara, F., Molesti, E. & Temperton, N. J. An Optimised Method for the Production using PEI, Titration and Neutralization of SARS-CoV Spike Luciferase Pseudotypes. *Bio-Protoc.* **7**, e2514 (2017). 34541175 10.21769/BioProtoc.2514 PMC8413606

[49] Pedregosa, F. et al. Scikit-learn: Machine Learning in Python. *J. Mach. Learn. Res.* **12**, 2825–2830 (2011).

[50] Hunter, J. D. Matplotlib: A 2D Graphics Environment. *Comput. Sci. Eng.* **9**, 90–95 (2007). 10.1109/MCSE.2007.55

[51] Schrödinger, L. & DeLano, W. PyMOL. (2020).

[52] Nutalai, R. et al. Potent cross-reactive antibodies following Omicron breakthrough in vaccinees. *Cell* **185**, 2116–2131.e18 (2022). 35662412 10.1016/j.cell.2022.05.014 PMC9120130

