# Supplementary material for: Computationally designed Spike antigens induce neutralising responses against the breadth of SARS-COV-2 variants
Source: NPJ Vaccines. 2024 Sep 9;9:164. doi: 10.1038/s41541-024-00950-9 (PMC11384739; doi:10.1038/s41541-024-00950-9)
Supplement: Supplementary file 1 — Supplementary Information [file 41541_2024_950_MOESM1_ESM.pdf]

Computationally designed Spike antigens induce neutralising responses against the breadth of SARS-COV-2 variants

Sneha Vishwanath[1], George Carnell[1], Martina Billmeier[2], Luis Ohlendorf[1], Patrick Neckermann[2], Benedikt Asbach[2], Charlotte George[1], Maria Suau Sans[1], Andrew Chan[1], Joey Olivier[1], Angalee Nadesalingam[1], Sebastian Einhauser[2], Nigel Temperton[3], Diego Cantoni[4], Joe Grove[4], Ingo Jordan[5], Volker Sandig[5], Paul Tonks[1], Johannes Geiger[6], Christian Dohmen[6], Verena Mummert [6], Anne Rosalind Samuel[6], Christian Plank[6], Rebecca Kinsley[1,7], Ralf Wagner[2,7,8], Jonathan Luke Heeney[1,7*].

Affiliations: [1]Lab of Viral Zoonotics, Department of Veterinary Medicine, University of Cambridge; Madingley Road, Cambridge, CB30ES, United Kingdom.
[2]Institute of Medical Microbiology and Hygiene, University of Regensburg, Regensburg, Germany.
[3]Viral Pseudotype Unit, Medway School of Pharmacy, The Universities of Kent and Greenwich at Medway, Chatham, United Kingdom.
[4]MRC-University of Glasgow Centre for Virus Research, United Kingdom
[5]ProBioGenAG, Berlin, Germany
[6]Ethris GmbH, Semmelweisstraße 3, 82152 Planegg, Germany
[7]DIOSynVax Ltd, University of Cambridge, Cambridge, United Kingdom.
[8]Institute of Clinical Microbiology and Hygiene, University Hospital Regensburg, Regensburg, Germany
*Corresponding author. Email: jlh66@cam.ac.uk

SUPPLEMENTARY FILE

Supplementary Note 1 : The consensus sequence of the spike protein used for generation of pseudo-viruses

>Beta
MFVFLVLLPLVSSQCVNLTTRTQLPPSYTNSFTRGVYYPDKVFRSSVLHSTQDLFLPFFS
NVTWFHAIHVSGTNGTKRFANPVLPFNDGVYFASTEKSNIIRGWIFGTTLDSKTQSLLIV
NNATNVVIKVCEFQFCNDPFLGVYYHKNNKSWMESEFRVYSSANNCTFEYVSQPFLMDLE
GKQGNFKNLREFVFKNIDGYFKIYSKHTPINLVRGLPQGFSALEPLVDLPIGINITRFQT
LHRSYLTPGDSSSGWTAGAAAYYVGYLQPRTFLLKYNENGTITDAVDCALDPLSETKCTL
KSFTVEKGIYQTSNFRVQPTESIVRFPNITNLCPFGEVFNATRFASVYAWNRKRISNCVA
DYSVLYNSASFSTFKCYGVSPTKLNDLCFTNVYADSFVIRGDEVRQIAPGQTGNIADYNY
KLPDDFTGCVIAWNSNNLDSKVGGNYNYLYRLFRKSNLKPFERDISTEIYQAGSTPCNGV
KGFNCYFPLQSYGFQPTYGVGYQPYRVVVLSFELLHAPATVCGPKKSTNLVKNKCVNFNF
NGLTGTGVLTESNKKFLPFQQFGRDIADTTDAVRDPQTLEILDITPCSFGGVSVITPGTN
TSNQVAVLYQGVNCTEVPVAIHADQLTPTWRVYSTGSNVFQTRAGCLIGAEHVNNSYECD
IPIGAGICASYQTQTNSPRRARSVASQSIIAYTMSLGVENSVAYSNNSIAIPTNFTISVT
TEILPVSMTKTSVDCTMYICGDSTECSNLLLQYGSFCTQLNRALTGIAVEQDKNTQEVFA
QVKQIYKTPPIKDFGGFNFSQILPDPSKPSKRSFIEDLLFNKVTLADAGFIKQYGDCLGD
IAARDLICAQKFNGLTVLPPLLTDEMIAQYTSALLAGTITSGWTFGAGAALQIPFAMQMA
YRFNGIGVTQNVLYENQKLIANQFNSAIGKIQDSLSSTASALGKLQDVVNQNAQALNTLV
KQLSSNFGAISSVLNDILSRLDKVEAEVQIDRLITGRLQSLQTYVTQQLIRAAEIRASAN
LAATKMSECVLGQSKRVDFCGKGYHLMSFPQSAPHGVVFLHVTYVPAQEKNFTTAPAICH
DGKAHFPREGVFVSNGTHWFVTQRNFYEPQIITTDNTFVSGNCDVVIGIVNNTVYDPLQP
ELDSFKEELDKYFKNHTSPDVDLGDISGINASVVNIQKEIDRLNEVAKNLNESLIDLQEL
GKYEQYIKWPWYIWLGFIAGLIAIVMVTIMLCCMTSCCSCLKGCCSCGSCCKFDEDDSEP
VLKGVKLHYT
>Gamma
MFVFLVLLPLVSSQCVNFTNRTQLPSAYTNSFTRGVYYPDKVFRSSVLHSTQDLFLPFFS
NVTWFHAIHVSGTNGTKRFDNPVLPFNDGVYFASTEKSNIIRGWIFGTTLDSKTQSLLIV
NNATNVVIKVCEFQFCNYPFLGVYYHKNNKSWMESEFRVYSSANNCTFEYVSQPFLMDLE
GKQGNFKNLSEFVFKNIDGYFKIYSKHTPINLVRDLPQGFSALEPLVDLPIGINITRFQT
LLALHRSYLTPGDSSSGWTAGAAAYYVGYLQPRTFLLKYNENGTITDAVDCALDPLSETK
CTLKSFTVEKGIYQTSNFRVQPTESIVRFPNITNLCPFGEVFNATRFASVYAWNRKRISN
CVADYSVLYNSASFSTFKCYGVSPTKLNDLCFTNVYADSFVIRGDEVRQIAPGQTGTIAD
YNYKLPDDFTGCVIAWNSNNLDSKVGGNYNYLYRLFRKSNLKPFERDISTEIYQAGSTPC
NGVKGFNCYFPLQSYGFQPTYGVGYQPYRVVVLSFELLHAPATVCGPKKSTNLVKNKCVN
FNFNGLTGTGVLTESNKKFLPFQQFGRDIADTTDAVRDPQTLEILDITPCSFGGVSVITP
GTNTSNQVAVLYQGVNCTEVPVAIHADQLTPTWRVYSTGSNVFQTRAGCLIGAEYVNNSY
ECDIPIGAGICASYQTQTNSPRRARSVASQSIIAYTMSLGAENSVAYSNNSIAIPTNFTI
SVTTEILPVSMTKTSVDCTMYICGDSTECSNLLLQYGSFCTQLNRALTGIAVEQDKNTQE
VFAQVKQIYKTPPIKDFGGFNFSQILPDPSKPSKRSFIEDLLFNKVTLADAGFIKQYGDC
LGDIAARDLICAQKFNGLTVLPPLLTDEMIAQYTSALLAGTITSGWTFGAGAALQIPFAM
QMAYRFNGIGVTQNVLYENQKLIANQFNSAIGKIQDSLSSTASALGKLQDVVNQNAQALN
TLVKQLSSNFGAISSVLNDILSRLDKVEAEVQIDRLITGRLQSLQTYVTQQLIRAAEIRA
SANLAAIKMSECVLGQSKRVDFCGKGYHLMSFPQSAPHGVVFLHVTYVPAQEKNFTTAPA
ICHDGKAHFPREGVFVSNGTHWFVTQRNFYEPQIITTDNTFVSGNCDVVIGIVNNTVYDP
LQPELDSFKEELDKYFKNHTSPDVDLGDISGINASFVNIQKEIDRLNEVAKNLNESLIDL
QELGKYEQYIKWPWYIWLGFIAGLIAIVMVTIMLCCMTSCCSCLKGCCSCGSCCKFDEDD
SEPVLKGVKLHYT
>Delta
MFVFLVLLPLVSSQCVNLRTRTQLPPAYTNSFTRGVYYPDKVFRSSVLHSTQDLFLPFFS
NVTWFHAIHVSGTNGTKRFDNPVLPFNDGVYFASIEKSNIIRGWIFGTTLDSKTQSLLIV
NNATNVVIKVCEFQFCNDPFLDVYYHKNNKSWMESGVYSSANNCTFEYVSQPFLMDLEGK
QGNFKNLREFVFKNIDGYFKIYSKHTPINLVRDLPQGFSALEPLVDLPIGINITRFQTLL
ALHRSYLTPGDSSSGWTAGAAAYYVGYLQPRTFLLKYNENGTITDAVDCALDPLSETKCT

LKSFTVEKGIYQTSNFRVQPTESIVRFPNITNLCPFGEVFNATRFASVYAWNRKRISNCV
ADYSVLYNSASFSTFKCYGVSPTKLNDLCFTNVYADSFVIRGDEVRQIAPGQTGKIADYN
YKLPDDFTGCVIAWNSNNLDSKVGGNYNYRYRLFRKSNLKPFERDISTEIYQAGSKPCNG
VEGFNCYFPLQSYGFQPTNGVGYQPYRVVVLSFELLHAPATVCGPKKSTNLVKNKCVNFN
FNGLTGTGVLTESNKKFLPFQQFGRDIADTTDAVRDPQTLEILDITPCSFGGVSVITPGT
NTSNQVAVLYQGVNCTEVPVAIHADQLTPTWRVYSTGSNVFQTRAGCLIGAEHVNNSYEC
DIPIGAGICASYQTQTNSRRRARSVASQSIIAYTMSLGAENSVAYSNNSIAIPTNFTISV
TTEILPVSMTKTSVDCTMYICGDSTECSNLLLQYGSFCTQLNRALTGIAVEQDKNTQEVF
AQVKQIYKTPPIKDFGGFNFSQILPDPSKPSKRSFIEDLLFNKVTLADAGFIKQYGDCLG
DIAARDLICAQKFNGLTVLPPLLTDEMIAQYTSALLAGTITSGWTFGAGAALQIPFAMQM
AYRFNGIGVTQNVLYENQKLIANQFNSAIGKIQDSLSSTASALGKLQNVVNQNAQALNTL
VKQLSSNFGAISSVLNDILSRLDKVEAEVQIDRLITGRLQSLQTYVTQQLIRAAEIRASA
NLAATKMSECVLGQSKRVDFCGKGYHLMSFPQSAPHGVVFLHVTYVPAQEKNFTTAPAIC
HDGKAHFPREGVFVSNGTHWFVTQRNFYEPQIITTDNTFVSGNCDVVIGIVNNTVYDPLQ
PELDSFKEELDKYFKNHTSPDVDLGDISGINASVVNIQKEIDRLNEVAKNLNESLIDLQE
LGKYEQYIKWPWYIWLGFIAGLIAIVMVTIMLCCMTSCCSCLKGCCSCGSCCKFDEDDSE
PVLKGVKLHYT

>BA.1

MFVFLVLLPLVSSQCVNLTTRTQLPPAYTNSFTRGVYYPDKVFRSSVLHSTQDLFLPFFS
NVTWFHVISGTNGTKRFDNPVLPFNDGVYFASIEKSNIIRGWIFGTTLDSKTQSLLIVNN
ATNVVIKVCEFQFCNDPFLDHKNNKSWMESEFRVYSSANNCTFEYVSQPFLMDLEGKQGN
FKNLREFVFKNIDGYFKIYSKHTPIIVREPEDLPQGFSALEPLVDLPIGINITRFQTLLA
LHRSYLTPGDSSSGWTAGAAAYYVGYLQPRTFLLKYNENGTITDAVDCALDPLSETKCTL
KSFTVEKGIYQTSNFRVQPTESIVRFPNITNLCPFDEVFNATRFASVYAWNRKRISNCVA
DYSVLYNLAPFFTFKCYGVSPTKLNDLCFTNVYADSFVIRGDEVRQIAPGQTGNIADYNY
KLPDDFTGCVIAWNSNKLDSKVSGNYNYLYRLFRKSNLKPFERDISTEIYQAGNKPCNGV
AGFNCYFPLRSYSFRPTYGVGHQPYRVVVLSFELLHAPATVCGPKKSTNLVKNKCVNFNF
NGLKGTGVLTESNKKFLPFQQFGRDIADTTDAVRDPQTLEILDITPCSFGGVSVITPGTN
TSNQVAVLYQGVNCTEVPVAIHADQLTPTWRVYSTGSNVFQTRAGCLIGAEYVNNSYECD
IPIGAGICASYQTQTKSHRRARSVASQSIIAYTMSLGAENSVAYSNNSIAIPTNFTISVT
TEILPVSMTKTSVDCTMYICGDSTECSNLLLQYGSFCTQLKRALTGIAVEQDKNTQEVFA
QVKQIYKTPPIKYFGGFNFSQILPDPSKPSKRSFIEDLLFNKVTLADAGFIKQYGDCLGD
IAARDLICAQKFKGLTVLPPLLTDEMIAQYTSALLAGTITSGWTFGAGAALQIPFAMQMA
YRFNGIGVTQNVLYENQKLIANQFNSAIGKIQDSLSSTASALGKLQDVVNHNAQALNTLV
KQLSSKFGAISSVLNDIFSRLDKVEAEVQIDRLITGRLQSLQTYVTQQLIRAAEIRASAN
LAATKMSECVLGQSKRVDFCGKGYHLMSFPQSAPHGVVFLHVTYVPAQEKNFTTAPAICH
DGKAHFPREGVFVSNGTHWFVTQRNFYEPQIITTDNTFVSGNCDVVIGIVNNTVYDPLQP
ELDSFKEELDKYFKNHTSPDVDLGDISGINASVVNIQKEIDRLNEVAKNLNESLIDLQEL
GKYEQYIKWPWYIWLGFIAGLIAIVMVTIMLCCMTSCCSCLKGCCSCGSCCKFDEDDSEP
VLKGVKLHYT

>BA.2

MFVFLVLLPLVSSQCVNLITRTQSYTNSFTRGVYYPDKVFRSSVLHSTQDLFLPFFSNVT
WFHAIHVSGTNGTKRFDNPVLPFNDGVYFASTEKSNIIRGWIFGTTLDSKTQSLLIVNNA
TNVVIKVCEFQFCNDPFLDVYYHKNNKSWMESEFRVYSSANNCTFEYVSQPFLMDLEGKQ
GNFKNLREFVFKNIDGYFKIYSKHTPINLGRDLPQGFSALEPLVDLPIGINITRFQTLLA
LHRSYLTPGDSSSGWTAGAAAYYVGYLQPRTFLLKYNENGTITDAVDCALDPLSETKCTL
KSFTVEKGIYQTSNFRVQPTESIVRFPNITNLCPFDEVFNATRFASVYAWNRKRISNCVA
DYSVLYNFAPFFAFKCYGVSPTKLNDLCFTNVYADSFVIRGNEVSQIAPGQTGNIADYNY
KLPDDFTGCVIAWNSNKLDSKVGGNYNYLYRLFRKSNLKPFERDISTEIYQAGNKPCNGV
AGFNCYFPLRSYGFRPTYGVGHQPYRVVVLSFELLHAPATVCGPKKSTNLVKNKCVNFNF
NGLTGTGVLTESNKKFLPFQQFGRDIADTTDAVRDPQTLEILDITPCSFGGVSVITPGTN
TSNQVAVLYQGVNCTEVPVAIHADQLTPTWRVYSTGSNVFQTRAGCLIGAEYVNNSYECD
IPIGAGICASYQTQTKSHRRARSVASQSIIAYTMSLGAENSVAYSNNSIAIPTNFTISVT
TEILPVSMTKTSVDCTMYICGDSTECSNLLLQYGSFCTQLKRALTGIAVEQDKNTQEVFA
QVKQIYKTPPIKYFGGFNFSQILPDPSKPSKRSFIEDLLFNKVTLADAGFIKQYGDCLGD

IAARDLICAQKFNGLTVLPPLLTDEMIAQYTSALLAGTITSGWTFGAGAALQIPFAMQMA
YRFNGIGVTQNVLYENQKLIANQFNSAIGKIQDSLSSTASALGKLQDVVNHNAQALNTLV
KQLSSKFGAISSVLNDILSRLDKVEAEVQIDRLITGRLQSLQTYVTQQLIRAAEIRASAN
LAATKMSECVLGQSKRVDFCGKGYHLMSFPQSAPHGVVFLHVTYVPAQEKNFTTAPAICH
DGKAHFPREGVFVSNGTHWFVTQRNFYEPQIITTDNTFVSGNCDVVIGIVNNTVYDPLQP
ELDSFKEELDKYFKNHTSPDVDLGDISGINASVVNIQKEIDRLNEVAKNLNESLIDLQEL
GKYEQYIKWPWYIWLGFIAGLIAIVMVTIMLCCMTSCCSCLKGCCSCGSCCKFDEDDSEP
VLKGVKLHYT
>BA.2.75.2
MFVFLVLLPLVSSQCVNLITRTQSYTNSFTRGVYYPDKVFRSSVLHSTQDLFLPFFSNVT
WFHAIHVSGTNGTKRFDNPVLPFNDGVYFASTEKSNIIRGWIFGTTLDSKTQSLLIVNNA
TNVVIKVCEFQFCNDPFLDVYYHENNKSRMESELRVYSSANNCTFEYVSQPFLMDLEGKQ
GNFKNLREFVFKNIDGYFKIYSKHTPVNLGRDLPQGFSALEPLVDLPIGINITRFQTLLA
LHRSYLTPGDSSSSWTAGAAAYYVGYLQPRTFLLKYNENGTITDAVDCALDPLSETKCTL
KSFTVEKGIYQTSNFRVQPTESIVRFPNITNLCPFHEVFNATTFASVYAWNRKRISNCVA
DYSVLYNFAPFFAFKCYGVSPTKLNDLCFTNVYADSFVIRGNEVRQIAPGQTGNIADYNY
KLPDDFTGCVIAWNSNNLDSKVGGNYNYLYRLFRKSNLKPFERDISTEIYQAGNKPCNGV
AGSNCYFPLQSYGFRPTYGVGHQPYRVVVLSFELLHAPATVCGPKKSTNLVKNKCVNFNF
NGLTGTGVLTESNKKFLPFQQFGRDIADTTDAVRDPQTLEILDITPCSFGGVSVITPGTN
TSNQVAVLYQGVNCTEVPVAIHADQLTPTWRVYSTGSNVFQTRAGCLIGAEYVNNSYECD
IPIGAGICASYQTQTKSHRRARSVASQSIIAYTMSLGAENSVAYSNNSIAIPTNFTISVT
TEILPVSMTKTSVDCTMYICGDSTECSNLLLQYGSFCTQLKRALTGIAVEQDKNTQEVFA
QVKQIYKTPPIKYFGGFNFSQILPDPSKPSKRSFIEDLLFNKVTLADAGFIKQYGDCLGD
IAARDLICAQKFNGLTVLPPLLTDEMIAQYTSALLAGTITSGWTFGAGAALQIPFAMQMA
YRFNGIGVTQNVLYENQKLIANQFNSAIGKIQDSLSSTASALGKLQDVVNHNAQALNTLV
KQLSSKFGAISSVLNDILSRLDKVEAEVQIDRLITGRLQSLQTYVTQQLIRAAEIRASAN
LAATKMSECVLGQSKRVDFCGKGYHLMSFPQSAPHGVVFLHVTYVPAQEKNFTTAPAICH
DGKAHFPREGVFVSNGTHWFVTQRNFYEPQIITTDNTFVSGNCDVVIGIVNNTVYDPLQP
ELDSFKEELDKYFKNHTSPDVDLGDISGINASVVNIQKEIDRLNEVAKNLNESLINLQEL
GKYEQYIKWPWYIWLGFIAGLIAIVMVTIMLCCMTSCCSCLKGCCSCGSCCKFDEDDSEP
VLKGVKLHYT
>BQ.1.1
MFVFLVLLPLVSSQCVNLITRTQSYTNSFTRGVYYPDKVFRSSVLHSTQDLFLPFFSNVT
WFHAISGTNGTKRFDNPVLPFNDGVYFASTEKSNIIRGWIFGTTLDSKTQSLLIVNNATN
VVIKVCEFQFCNDPFLDVYYHKNNKSWMESEFRVYSSANNCTFEYVSQPFLMDLEGKQGN
FKNLREFVFKNIDGYFKIYSKHTPINLGRDLPQGFSALEPLVDLPIGINITRFQTLLALH
RSYLTPGDSSSGWTAGAAAYYVGYLQPRTFLLKYNENGTITDAVDCALDPLSETKCTLKS
FTVEKGIYQTSNFRVQPTESIVRFPNITNLCPFDEVFNATTFASVYAWNRKRISNCVADY
SVLYNFAPFFAFKCYGVSPTKLNDLCFTNVYADSFVIRGNEVSQIAPGQTGNIADYNYKL
PDDFTGCVIAWNSNKLDSTVGGNYNYRYRLFRKSKLKPFERDISTEIYQAGNKPCNGVAG
VNCYFPLQSYGFRPTYGVGHQPYRVVVLSFELLHAPATVCGPKKSTNLVKNKCVNFNFNG
LTGTGVLTESNKKFLPFQQFGRDIADTTDAVRDPQTLEILDITPCSFGGVSVITPGTNTS
NQVAVLYQGVNCTEVPVAIHADQLTPTWRVYSTGSNVFQTRAGCLIGAEYVNNSYECDIP
IGAGICASYQTQTKSHRRARSVASQSIIAYTMSLGAENSVAYSNNSIAIPTNFTISVTTE
ILPVSMTKTSVDCTMYICGDSTECSNLLLQYGSFCTQLKRALTGIAVEQDKNTQEVFAQV
KQIYKTPPIKYFGGFNFSQILPDPSKPSKRSFIEDLLFNKVTLADAGFIKQYGDCLGDIA
ARDLICAQKFNGLTVLPPLLTDEMIAQYTSALLAGTITSGWTFGAGAALQIPFAMQMAYR
FNGIGVTQNVLYENQKLIANQFNSAIGKIQDSLSSTASALGKLQDVVNHNAQALNTLVKQ
LSSKFGAISSVLNDILSRLDKVEAEVQIDRLITGRLQSLQTYVTQQLIRAAEIRASANLA
ATKMSECVLGQSKRVDFCGKGYHLMSFPQSAPHGVVFLHVTYVPAQEKNFTTAPAICHDG
KAHFPREGVFVSNGTHWFVTQRNFYEPQIITTDNTFVSGNCDVVIGIVNNTVYDPLQPEL
DSFKEELDKYFKNHTSPDVDLGDISGINASVVNIQKEIDRLNEVAKNLNESLIDLQELGK
YEQYIKWPWYIWLGFIAGLIAIVMVTIMLCCMTSCCSCLKGCCSCGSCCKFDEDDSEPVL
KGVKLHYT
>BA.2.86

MFVFLVLLPLVSSQCVMPLFNLITTTQSYTNSFTRGVYYPDKVFRSSVLHLTQDLFLPFF
SNVTWFHAISGTNGTKRFDNPVLPFNDGVYFASTEKSNIIRGWIFGTTLDSKTQSLLIVN
NATNVFIKVCEFQFCNDPFLDVYHKNNKSWMESESGVYSSANNCTFEYVSQPFLMDLEGK
QGNFKNLREFVFKNIDGYFKIYSKHTPIIGRDFPQGFSALEPLVDLPIGINITRFQTLLA
LNRSYLTPGDSSSGWTAGAADYYVGYLQPRTFLLKYNENGTITDAVDCALDPLSETKCTL
KSFTVEKGIYQTSNFRVQPTESIVRFPNVTNLCPFHEVFNATRFASVYAWNRTRISNCVA
DYSVLYNFAPFFAFKCYGVSPTKLNDLCFTNVYADSFVIKGNEVSQIAPGQTGNIADYNY
KLPDDFTGCVIAWNSNKLDSKHSGNYDYWYRLFRKSKLKPFERDISTEIYQAGNKPCKGK
GPNCYFPLQSYGFRPTYGVGHQPYRVVVLSFELLHAPATVCGPKKSTNLVKNKCVNFNFN
GLTGTGVLTKSNKKFLPFQQFGRDIVDTTDAVRDPQTLEILDITPCSFGGVSVITPGTNT
SNQVAVLYQGVNCTEVSVAIHADQLTPTWRVYSTGSNVFQTRAGCLIGAEYVNNSYECDI
PIGAGICASYQTQTKSRRRARSVASQSIIAYTMSLGAENSVAYSNNSIAIPTNFTISVTT
EILPVSMTKTSVDCTMYICGDSTECSNLLLQYGSFCTQLKRALTGIAVEQDKNTQEVFAQ
VKQIYKTPPIKYFGGFNFSQILPDPSKPSKRSFIEDLLFNKVTLADAGFIKQYGDCLGDI
AARDLICAQKFNGLTVLPPLLTDEMIAQYTSALLAGTITSGWTFGAGAALQIPFAMQMAY
RFNGIGVTQNVLYENQKLIANQFNSAIGKIQDSLFSTASALGKLQDVVNHNAQALNTLVK
QLSSKFGAISSVLNDILSRLDKVEAEVQIDRLITGRLQSLQTYVTQQLIRAAEIRASANL
AATKMSECVLGQSKRVDFCGKGYHLMSFPQSAPHGVVFLHVTYVPAQEKNFTTAPAICHD
GKAHFPREGVFVSNGTHWFVTQRNFYEPQIITTDNTFVSGNCDVVIGIVNNTVYDPLQLE
LDSFKEELDKYFKNHTSPDVDLGDISGINASVVNIQKEIDRLNEVAKNLNESLIDLQELG
KYEQYIKWPWYIWLGFIAGLIAIVMVTIMLCCMTSCCSCLKGCCSCGSCCKFDEDDSEPV
LKGVKLHYT

>XBB.1.5

MFVFLVLLPLVSSQCVNLITRTQSYTNSFTRGVYYPDKVFRSSVLHSTQDLFLPFFSNVT
WFHAIHVSGTNGTKRFDNPALPFNDGVYFASTEKSNIIRGWIFGTTLDSKTQSLLIVNNA
TNVVIKVCEFQFCNDPFLDVYQKNNKSWMESEFRVYSSANNCTFEYVSQPFLMDLEGKEG
NFKNLREFVFKNIDGYFKIYSKHTPINLERDLPQGFSALEPLVDLPIGINITRFQTLLAL
HRSYLTPVDSSSGWTAGAAAYYVGYLQPRTFLLKYNENGTITDAVDCALDPLSETKCTLK
SFTVEKGIYQTSNFRVQPTESIVRFPNITNLCPFHEVFNATTFASVYAWNRKRISNCVAD
YSVIYNFAPFFAFKCYGVSPTKLNDLCFTNVYADSFVIRGNEVSQIAPGQTGNIADYNYK
LPDDFTGCVIAWNSNKLDSKPSGNYNYLYRLFRKSKLKPFERDISTEIYQAGNKPCNGVA
GPNCYSPLQSYGFRPTYGVGHQPYRVVVLSFELLHAPATVCGPKKSTNLVKNKCVNFNFN
GLTGTGVLTESNKKFLPFQQFGRDIADTTDAVRDPQTLEILDITPCSFGGVSVITPGTNT
SNQVAVLYQGVNCTEVPVAIHADQLTPTWRVYSTGSNVFQTRAGCLIGAEYVNNSYECDI
PIGAGICASYQTQTKSHRRARSVASQSIIAYTMSLGAENSVAYSNNSIAIPTNFTISVTT
EILPVSMTKTSVDCTMYICGDSTECSNLLLQYGSFCTQLKRALTGIAVEQDKNTQEVFAQ
VKQIYKTPPIKYFGGFNFSQILPDPSKPSKRSFIEDLLFNKVTLADAGFIKQYGDCLGDI
AARDLICAQKFNGLTVLPPLLTDEMIAQYTSALLAGTITSGWTFGAGAALQIPFAMQMAY
RFNGIGVTQNVLYENQKLIANQFNSAIGKIQDSLSSTASALGKLQDVVNHNAQALNTLVK
QLSSKFGAISSVLNDILSRLDKVEAEVQIDRLITGRLQSLQTYVTQQLIRAAEIRASANL
AATKMSECVLGQSKRVDFCGKGYHLMSFPQSAPHGVVFLHVTYVPAQEKNFTTAPAICHD
GKAHFPREGVFVSNGTHWFVTQRNFYEPQIITTDNTFVSGNCDVVIGIVNNTVYDPLQPE
LDSFKEELDKYFKNHTSPDVDLGDISGINASVVNIQKEIDRLNEVAKNLNESLIDLQELG
KYEQYIKWPWYIWLGFIAGLIAIVMVTIMLCCMTSCCSCLKGCCSCGSCCKFDEDDSEPV
LKGVKLHYT

>XBB

MFVFLVLLPLVSSQCVNLITRTQLPPSYTNSFTRGVYYPDKVFRSSVLHSTQDLFLPFFS
NVTWFHAIHVSGTNGTKRFDNPALPFNDGVYFASTEKSNIIRGWIFGTTLDSKTQSLLIV
NNATNVVIKVCEFQFCNDPFLDVYYQKNNKSWMESEFRVYSSANNCTFEYVSQPFLMDLE
GKEGNFKNLREFVFKNIDGYFKIYSKHTPINLERDLPQGFSALEPLVDLPIGINITRFQT
LLALHRSYLTPGDSSSGWTAGAAAYYVGYLQPRTFLLKYNENGTITDAVDCALDPLSETK
CTLKSFTVEKGIYQTSNFRVQPTESIVRFPNITNLCPFHEVFNATTFASVYAWNRKRISN
CVADYSVIYNFAPFFAFKCYGVSPTKLNDLCFTNVYADSFVIRGNEVSQIAPGQTGNIAD
YNYKLPDDFTGCVIAWNSNKLDSKPSGNYNYLYRLFRKSKLKPFERDISTEIYQAGNKPC
NGVAGSNCYSPLQSYGFRPTYGVGHQPYRVVVLSFELLHAPATVCGPKKSTNLVKNKCVN

```
FNFNGLTGTGVLTESNKKFLPFQQFGRDIADTTDAVRDPQTLEILDITPCSFGGVSVITP
GTNTSNQVAVLYQGVNCTEVPVAIHADQLTPTWRVYSTGSNVFQTRAGCLIGAEYVNNSY
ECDIPIGAGICASYQTQTKSHRRARSVASQSIIAYTMSLGAENSVAYSNNSIAIPTNFTI
SVTTEILPVSMTKTSVDCTMYICGDSTECSNLLLQYGSFCTQLKRALTGIAVEQDKNTQE
VFAQVKQIYKTPPIKYFGGFNFSQILPDPSKPSKRSFIEDLLFNKVTLADAGFIKQYGDC
LGDIAARDLICAQKFNGLTVLPPLLTDEMIAQYTSALLAGTITSGWTFGAGAALQIPFAM
QMAYRFNGIGVTQNVLYENQKLIANQFNSAIGKIQDSLSSTASALGKLQDVVNHNAQALN
TLVKQLSSKFGAISSVLNDILSRLDKVEAEVQIDRLITGRLQSLQTYVTQQLIRAAEIRA
SANLAATKMSECVLGQSKRVDFCGKGYHLMSFPQSAPHGVVFLHVTYVPAQEKNFTTAPA
ICHDGKAHFPREGVFVSNGTHWFVTQRNFYEPQIITTDNTFVSGNCDVVIGIVNNTVYDP
LQPELDSFKEELDKYFKNHTSPDVDLGDISGINASVVNIQKEIDRLNEVAKNLNESLIDL
QELGKYEQYIKWPWYIWLGFIAGLIAIVMVTIMLCCMTSCCSCLKGCCSCGSCCKFDEDD
SEPVLKGVKLHYT
```
